# Inhibition of H1N1 influenza virus infection by zinc oxide nanoparticles: another emerging application of nanomedicine

**DOI:** 10.1186/s12929-019-0563-4

**Published:** 2019-09-10

**Authors:** Hadi Ghaffari, Ahmad Tavakoli, Abdolvahab Moradi, Alijan Tabarraei, Farah Bokharaei-Salim, Masoumeh Zahmatkeshan, Mohammad Farahmand, Davod Javanmard, Seyed Jalal Kiani, Maryam Esghaei, Vahid Pirhajati-Mahabadi, Angila Ataei-Pirkooh, Seyed Hamidreza Monavari

**Affiliations:** 1 Department of Medical Virology, Iran University of Medical Sciences, Tehran, Iran; 2 Department of Microbiology, School of Medicine, Golestan University of Medical Sciences, Gorgan, Iran; 3 Cellular and Molecular Research Center, Iran University of Medical Sciences, Tehran, Iran; 4 Department of Medical Nanotechnology, Faculty of Advanced Technologies in Medicine, Iran University of Medical Sciences, Tehran, Iran; 5 Department of Virology, School of Public Health, Tehran University of Medical Sciences, Tehran, Iran; 6 Neuroscience Research Center, Iran University of Medical Sciences, Tehran, Iran

**Keywords:** Antiviral activity, Zinc oxide nanoparticle, H1N1 influenza, Polyethylene glycol

## Abstract

**Background:**

Currently available anti-influenza drugs are often associated with limitations such as toxicity and the appearance of drug-resistant strains. Therefore, there is a pressing need for the development of novel, safe and more efficient antiviral agents. In this study, we evaluated the antiviral activity of zinc oxide nanoparticles (ZnO-NPs) and PEGylated zinc oxide nanoparticles against H1N1 influenza virus.

**Methods:**

The nanoparticles were characterized using the inductively coupled plasma mass spectrometry, x-ray diffraction analysis, and electron microscopy. MTT assay was applied to assess the cytotoxicity of the nanoparticles, and anti-influenza activity was determined by TCID50 and quantitative Real-Time PCR assays. To study the inhibitory impact of nanoparticles on the expression of viral antigens, an indirect immunofluorescence assay was also performed.

**Results:**

Post-exposure of influenza virus with PEGylated ZnO-NPs and bare ZnO-NPs at the highest non-toxic concentrations could be led to 2.8 and 1.2 log10 TCID50 reduction in virus titer when compared to the virus control, respectively (*P* < 0.0001). At the highest non-toxic concentrations, the PEGylated and unPEGylated ZnO-NPs led to inhibition rates of 94.6 and 52.2%, respectively, which were calculated based on the viral loads. There was a substantial decrease in fluorescence emission intensity in viral-infected cell treated with PEGylated ZnO-NPs compared to the positive control.

**Conclusions:**

Taken together, our study indicated that PEGylated ZnO-NPs could be a novel, effective, and promising antiviral agent against H1N1 influenza virus infection, and future studies can be designed to explore the exact antiviral mechanism of these nanoparticles.

## Background

Influenza viruses are important human respiratory tract pathogens responsible for the seasonal epidemics and sporadic pandemics around the world [1]. According to the recent estimates reported by the World Health Organization (WHO), seasonal influenza epidemics lead to about 3–5 million cases of severe illness and approximately 290.000 to 650.000 deaths annually worldwide [2]. Influenza viruses are classified as type A, B, C and D on the basis of antigenicity of the viral nucleoprotein and major matrix protein, of which only influenza A and B viruses are the major culprit in human disease [3–6].

Presently, there are only two classes of drugs available against different influenza A strains and subtypes licensed by the U.S. Food and Drug Administration (FDA): matrix-2 (M2) protein ion channel blockers (such as amantadine and rimantadine) and neuraminidase (NA) inhibitors (such as zanamivir and oseltamivir) [7]. However, during the last years, there has been a remarkable increase in the emergence of drug-resistant strains, which have become a major public health concern around the world [8, 9]. Therefore, there is a growing need to identify and evaluate alternative anti-influenza agents which exert a different mechanism of action compared with the conventional drugs.

Recent advancements in nanotechnology have provided unique opportunities in the field of drug development programs. Nanoparticles are known as major products of the nanotechnologies with at least one dimension of 100 nm or less, and have attracted great interest due to their intriguing and unique properties compared to their bulk material [10, 11]. These characteristics make them suitable for different biomedical applications such as drug delivery, medical diagnostics and therapeutics [12]. Different biological and antimicrobial properties can also be achieved by surface modifications of nanoparticles [13].

During the last years, metal nanoparticles have been shown to be efficient against a wide range of pathogens including bacteria, fungi, parasites and viruses [14]. Among metal nanoparticles, zinc oxide nanoparticles (ZnO-NPs) have been demonstrated to exert antimicrobial activities against various human pathogens [15]. However, most studies have focused on their inhibitory actions on bacterial infections, and there is limited studies evaluating the interaction between ZnO-NPs and viruses. In a recent work by our group, we found a strong inhibitory effects of ZnO-NPs and polyethylene glycol (PEG)-coated ZnO-NPs (ZnO-PEG-NPs) on HSV-1 [16]. In this line, we have decided to conduct the current study to investigate the effects of ZnO-NPs and ZnO-PEG-NPs on the replication of H1N1 influenza virus, which are amongst the most challenging viruses that threaten human health.

## Methods

### Preparation and characterization of nanoparticles

The powdered zinc oxide nanoparticles were purchased from US Research Nanomaterials (USA; Product Number: US3590). Nanoparticles were suspended in Dulbecco’s Modified Eagle’s medium (DMEM) (Invitrogen, USA) and the suspension were subjected to sonication to prevent agglomeration and make different concentrations. Oseltamivir purchased from Sigma-Aldrich (St. Louis, MO, USA) were dissolved in DMEM and used as a standard drug against influenza at different concentrations. Polyethylene glycol (PEG) 6000 was also purchased from Sigma-Aldrich. PEGylated ZnO-NPs were synthesized by mechanical method, as described in detail previously [16]. Inductively coupled plasma mass spectrometry (ICP-MS), X-ray diffraction analysis (XRD), Transmission Electron Microscopy (TEM), and Field Emission Scanning Electron Microscope (FE-SEM) were used for characterization of nanoparticles. Thermogravimetric analysis (TGA) was also performed to demonstrate the presence of PEG on the surface of ZnO nanoparticles [16].

### Cell culture and virus propagation

Madin-Darby canine kidney (MDCK)-SIAT1 cells were a gift from the Razi Vaccine and Serum Research Institute (Karaj, Iran). The cells were grown at 37 °C in 5% CO2 in DMEM, supplemented with 10% heat-inactivated fetal bovine serum (FBS; Invitrogen, USA), 1 mM sodium pyruvate (Merck, Germany), 2 mM L-glutamine (Merck, Germany), and 100 U/ml penicillin and 100 μg/ml streptomycin sulfate (Sigma-Aldrich, USA).

Influenza A/Puerto Rico/8/34 (H1N1; PR8) was also obtained from the Razi Vaccine and Serum Research Institute, and propagated in MDCK-SIAT1 cells. For virus-stock preparation, MDCK-SIAT1 cell monolayer in 25-cm^2^ flask (SPL Life Science, South Korea) was washed three times with phosphate-buffered saline (PBS, Bio-Idea, Iran), and the cells were infected with the virus at a multiplicity of infection (MOI) of 0.1 for 1 h at 35 °C. Afterwards, the virus inoculum was removed and the cells were overlaid with infection medium containing serum-free DMEM, 2 μg/ml trypsin-TPCK (Merck, Germany), 25 mM HEPES buffer (Sigma-Aldrich, USA), and 0.14% of bovine serum albumin (BSA; Sigma-Aldrich, USA), and the flask was then incubated at 35 °C for an additional 48 h. The virus-containing supernatants were harvested at 48 h post infection, clarified by centrifugation at 2500 rpm for 10 min at 4°, and filtered by sterile syringe filter 0.22 μm (Millipore, Ireland). The virus was then aliquoted into sterile cryovials and stored frozen at − 80 °C until use. Virus was titrated using the tissue culture infectious dose 50 (TCID50) method according to the Reed and Muench formula [17], and was used for the next in vitro experiments at the titer of 100 TCID50/mL.

### Determination of cell viability

The cytotoxicity of nanoparticles was assessed by MTT assay. Briefly, MDCK-SIAT1 cells at a density of 1 × 10^5^ cells/mL were seeded in a flat-bottomed 96-well microtiter plate (SPL Life Science, South Korea), and were incubated for 24 h at 37 °C and 5% CO2. A range of concentrations from 25 to 225 μg/ml of nanoparticles was prepared using the cell culture medium and was added to the plate in triplicate. After 48 h, the treatments were removed, and 10 μL of MTT reagent and 100 μL RPMI (Bio-Idea, Iran) were added to each well and incubated for a further 4 h. The medium was then removed and 50 μL of DMSO solution was added to the wells. Finally, the plate was read at 550 nm by a microplate reader (Hiperion MPR 4+, Germany).

### Assessment of antiviral activities

#### Virucidal activity

To evaluate direct effects of ZnO and ZnO-PEG nanoparticles on H1N1 influenza particles, equal volumes of the viral suspensions (100 TCID50/ml) and nanoparticles suspensions in non-toxic concentration ranges were mixed and incubated at 37 °C for 4 h in a humidified 5% CO2 atmosphere. The mixture (100 μL) was then added in triplicated wells of the confluent monolayer of MDCK-SIAT1 cells (2 × 10^4^ cells/well) in a flat-bottomed 96-well microtiter plate and further incubated for 1 h at 35 °C. The virus control (infected but untreated) and cell control (uninfected untreated cells) were kept in each plate prepared throughout the experiment. After 1 h incubation, the mixture was discarded, and the cells were washed three times with PBS to remove non-absorbed viruses and overlaid with infection medium. The plate was then incubated for 48 h at 35 °C in a humidified 5% CO2 atmosphere.

#### Pre-exposure antiviral activity

The confluent monolayer of MDCK-SIAT1 cells (2 × 10^4^ cells/well) in a flat-bottomed 96-well microtiter plate were pre-incubated with different concentrations of ZnO and ZnO-PEG nanoparticles in non-toxic concentration ranges in triplicates for 3 h at 37 °C. The media containing nanoparticles was discarded from the wells, and the cells were washed three times with PBS and then incubated for 1 h at 35 °C with 100 TCID50/mL virus. Afterwards, the virus inocula were removed from the wells, and the cells were washed three times with PBS, and were then overlaid with infection medium. The plate was further incubated for 48 h at 35 °C in a humidified 5% CO2 atmosphere. The virus and cell controls were kept as described above.

### Cell co-treatment assay

The co-treatment assay was performed to evaluate the functions of nanoparticles in inhibiting viral binding. MDCK-SIAT1 cells were grown in a flat-bottomed 96-well microtiter plate at the density of 2 × 10^4^ cells/well. The media was removed from all wells and 100 μL of nanoparticles suspensions at their non-toxic concentrations and 100 μL of 100 TCID50/mL viral suspensions were added simultaneously to the cells in triplicated and incubated for 1 h at 35 °C. The virus and cell controls were also included in this assay. Following 1 h incubation, the solution on the cells was discarded and the cells were washed three times with PBS, and were overlaid with infection medium. The plate was incubated for an additional 48 h at 35 °C with 5% CO2.

### Post-exposure antiviral activity

The confluent monolayer of MDCK-SIAT1 cells (2 × 10^4^ cells/well) in all wells of a flat-bottomed 96-well microtiter plate were incubated with 100 μL of 100 TCID50/mL H1N1 virus suspensions for 1 h at 35 °C in a humidified 5% CO2 incubator. The virus inocula were then discarded from the wells, and the cells were washed three times with PBS for removing unattached viruses. Different non-cytotoxic concentrations of ZnO and ZnO-PEG nanoparticles suspended in infection medium were then added in triplicate to the wells and the plate further incubated for 48 h at 35 °C in a humidified 5% CO2 atmosphere. The virus control (virus + DMEM) and the cell control (uninfected cells in DMEM) were also included in this experiment. This assay was also carried out for oseltamivir and soluble polyethylene glycol.

At the indicated time of all above experiments (at 48 h), the supernatant of each well was harvested and was subjected to TCID50 and quantitative Real-Time PCR assays to determine the amount of total progeny virus.

### Quantitative Real-Time PCR assay

To determine influenza viral load, a quantitative system using Real-Time PCR assay was carried out. Total RNA was extracted from the supernatants using the AccuPrep® Viral RNA Extraction Kit (Bioneer, South Korea), based on manufacturer’s protocol. The extracted RNA was then subjected to reverse transcription using the cDNA Synthesis Kit (Roche Diagnostics, Germany), according to manufacturer’s recommendations. Finally, quantitative Real-Time PCR was performed using the Genesig® Advanced kit (PrimerDesign Ltd., United Kingdom), according to manufacturer’s instructions. The kit contains primers and probe designed for detection of all influenza A subtypes. The assay was performed using the Rotor-Gene Q instrument (Qiagen, Germany) under the following conditions: 5 min activation of Taq DNA polymerase at 95 °C, followed by 40 cycles of 10s at 95 °C and 60s at 60 °C.

### Indirect immunofluorescence assay (IFA)

MDCK-SIAT1 cells (5.0 × 10^4^) were seeded on sterile glass coverslips (Nunc, Denmark) in a 24-well plate and grown until 80–90% confluence. The media was discarded and the cells were incubated with 200 μL of 100 TCID50/mL H1N1 virus suspensions for 1 h at 35 °C in a humidified 5% CO2 incubator. The virus inocula were then discarded from the wells, and the cells were washed three times with PBS. The maximum non-cytotoxic concentrations of nanoparticles (75 and 200 μg/ml of ZnO-NPs and ZnO-PEG-NPs, respectively) suspended in serum-free DMEM supplemented with trypsin-TPCK, HEPES buffer, and BSA were then added to the wells and the plate was incubated at 35 °C with 5% CO2. The virus and cell controls were also included in this experiment. After 24 h, the cells were fixed with cold acetone (4 °C) for 20 min, and the fixed cells were overlaid with anti-influenza A monoclonal antibody (Chemicon-Millipore, USA), followed by incubation at 37 °C for 45 min. In the next step, the cells were washed three time with PBS, and were then overlaid with fluorescein isothiocyanate (FITC)-conjugated mouse anti-human antibody (Dako, Germany), followed by incubation at 37 °C for 30 min. Afterwards, the cells were washed three time with PBS and coverslips were mounted in slides with glycerol buffer. Ultimately, the cells were visualized under the Olympus BH2-RFCA fluorescence microscope (Tokyo, Japan).

### Statistical analysis

Values represent the mean of three independent experiments. The results were tabulated, and differences between means were statistically analyzed using one-way analysis of variance (ANOVA), followed by Tukey’s multiple comparison test. All analyses were carried out using the GraphPad Prism software, version 7.0 (GraphPad Software, USA), and *P* values less than 0.05 were taken as statistically significant.

## Results

### Characterization of the nanoparticles

**Fig. 1:**
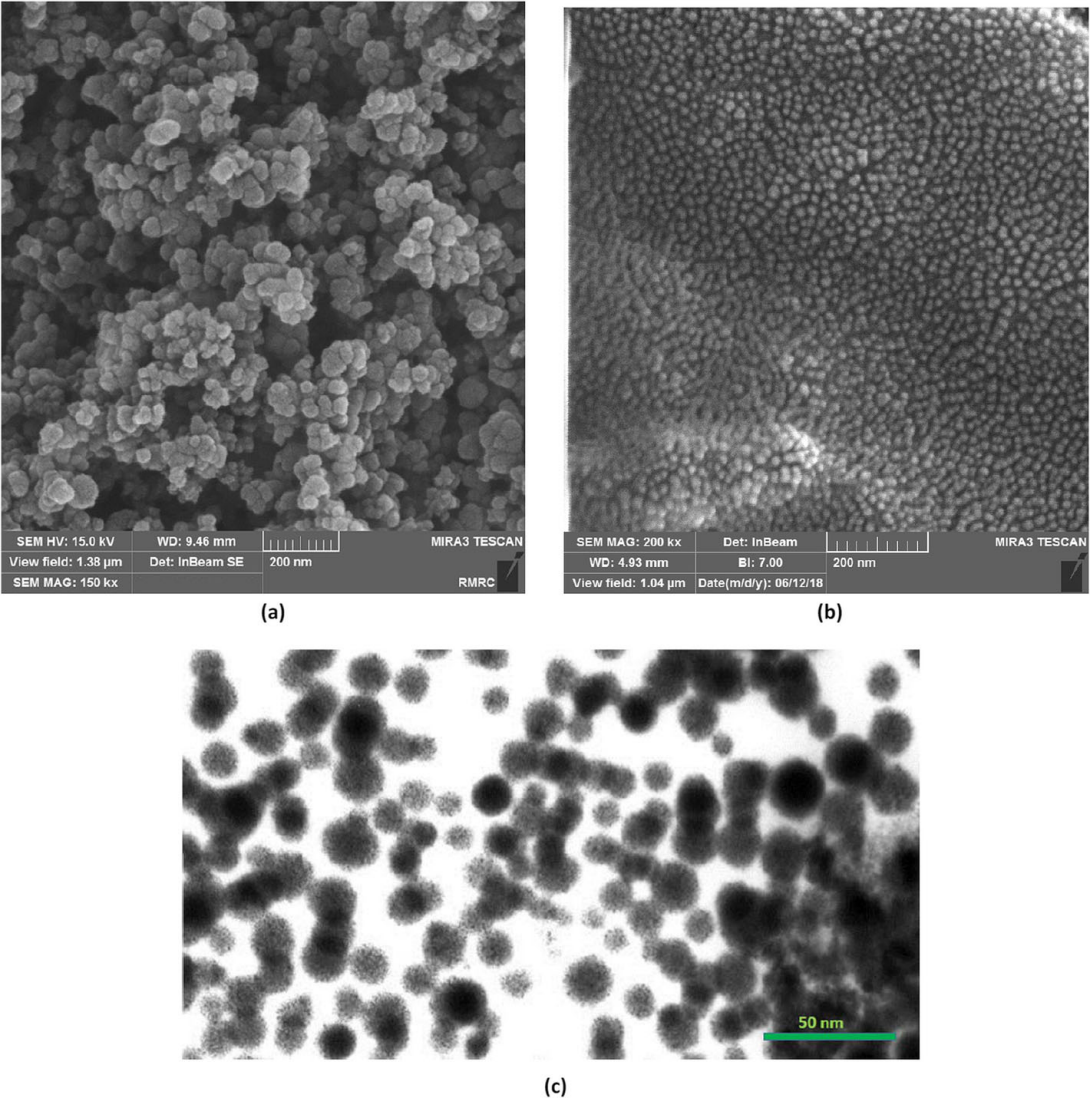
FE-SEM images of ZnO-NPs (**a**) and ZnO-PEG-NPs (**b**); TEM image of ZnO-PEG-NPs (**c**)

The FE-SEM images of ZnO-NPs and ZnO-PEG-NPs are shown in Fig. 1. The average diameters of ZnO-NPs ranged between 20 and 50 nm, whereas the ZnO-PEG-NPs were ranged from 16 to 20 nm. This reveals that PEGylation of ZnO-NPs by severe ball milling technique has led to a substantial decrease in the size of nanoparticles. The both nanoparticles were also spherical shaped and uniform. Surface coating of ZnO-NPs was also observed in Fig. 1 (c).

**Fig. 2:**
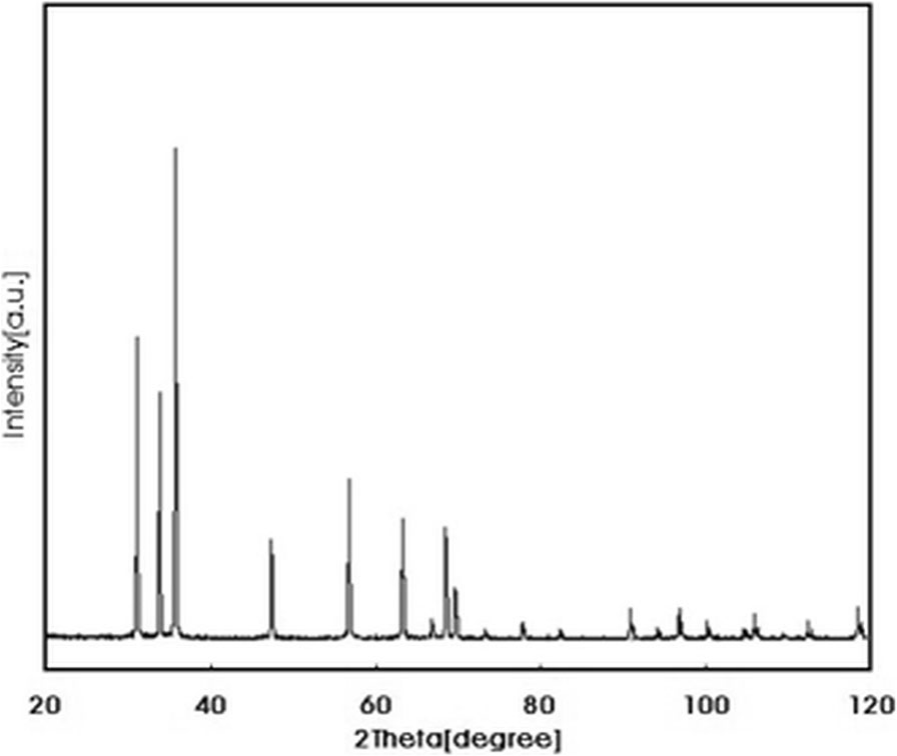
Powder X-ray Diffraction Pattern of ZnO-NPs

Figure 2 indicates the XRD powder diffraction patterns of the ZnO-NPs. The position and relative intensities of all diffraction peaks are similar to the standard XRD pattern of ZnO [18, 19].

**Fig. 3:**
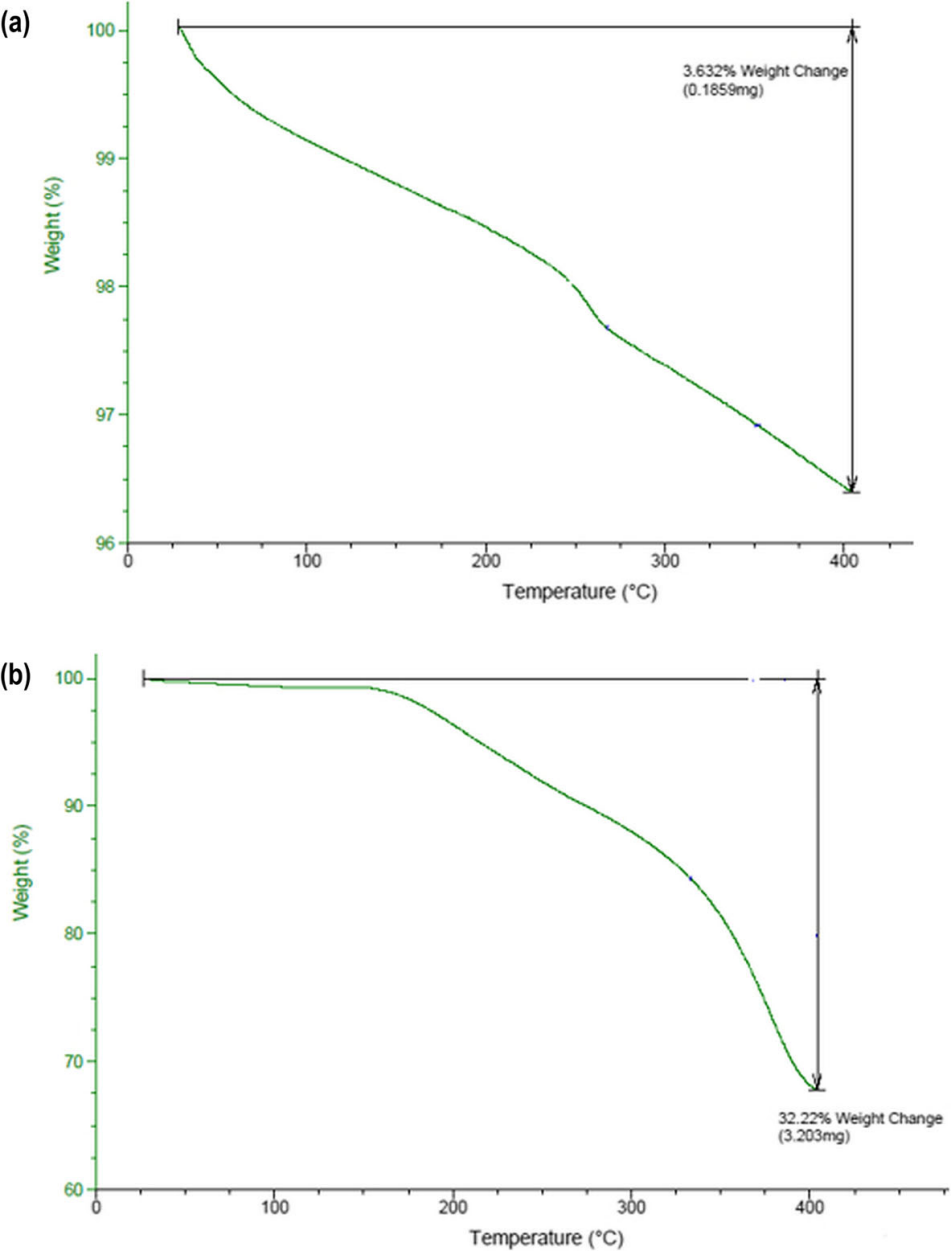
Thermogravimetric analysis: **a**) unPEGylated ZnO-NPs; **b**) PEGylated ZnO-NPs

In addition, ICP-MS measurement confirmed the high purity level of ZnO-NPs. The thermogravimetric analysis (TGA) of the ZnO-NPs and ZnO-PEG-NPs is presented in Fig. 3. The ZnO-PEG-NPs showed a significant weight loss of 32.22% at a temperature of 400 °C, whereas the ZnO-NPs showed a small weight loss of 3.6% at the same temperature. This corresponds to loss of polyethylene glycol, which was coated on the surface of ZnO-NPs.

### Cytotoxicity assay

**Fig. 4:**
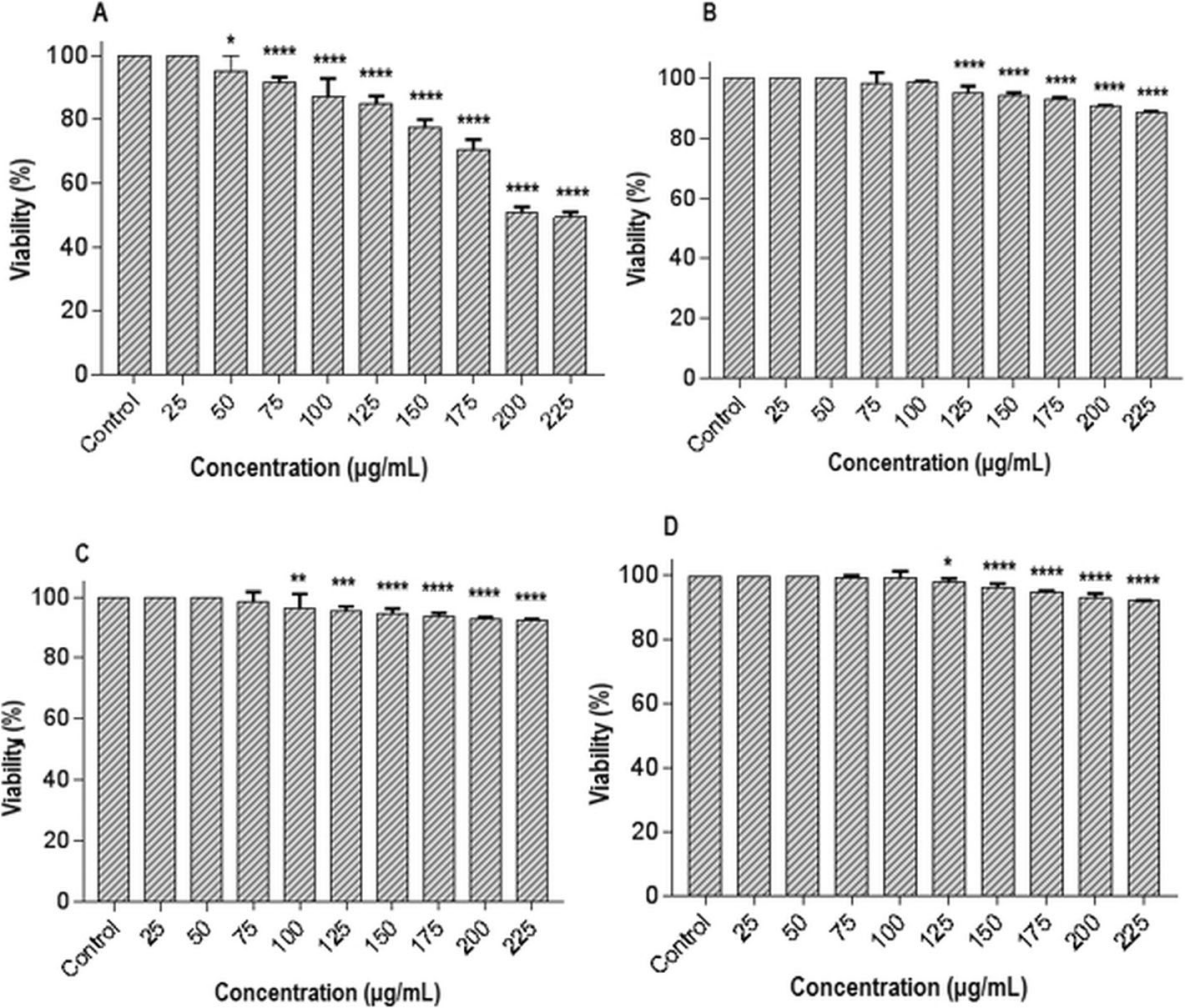
Cytotoxicity of ZnO-NPs (**a**), ZnO-PEG-NPs (**b**), polyethylene glycol (**c**), and oseltamivir (**d**) on MDCK-SIAT1 cells. * Statistically significant (*p* < 0.05). ** Statistically significant (*p* = 0.003). ** Statistically significant (*p* = 0.0005). **** Highly statistically significant (*p* = 0.0001). Error bars represent the confidence interval for the mean (*n* = 3) at the 95% level

Cytotoxic effects of ZnO-NPs, ZnO-PEG-NPs, polyethylene glycol, and oseltamivir on MDCK-SIAT1 cells were determined using the MTT assay. As shown in Fig. 4, polyethylene glycol and oseltamivir did not show significant cytotoxic effects toward MDCK-SIAT1 cells. The results obtained in the MTT assay revealed that the cytotoxicity of ZnO-PEG-NPs was significantly lower than that of ZnO-NPs, so that the viability was determined greater than 90% up to the concentration of 75 and 200 μg/mL of ZnO-NPs and ZnO-PEG-NPs, respectively.

### Assessment of antiviral activity

The results of TCID50 assay showed that the pre- and co-exposure of cells to ZnO-NPs and ZnO-PEG-NPs did not lead to any reduction of the H1N1 influenza virus titer. Meanwhile, virucidal activity was not observed at any concentrations of nanoparticles, suggesting that nanoparticles could not act directly against the influenza virus particle resulting in viral inactivation.

**Fig. 5:**
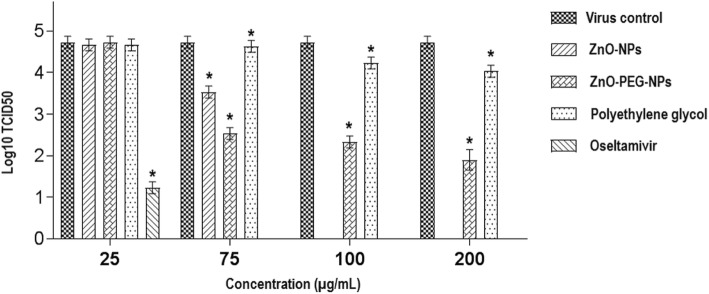
Assessment of the post-exposure antiviral activity of ZnO-NPs, ZnO-PEG-NPs, polyethylene glycol, and oseltamivir on the titer of H1N1 influenza virus by TCID50 assay. * Statistically significant (*p* < 0.0001). Error bars represent the confidence interval for the mean (*n* = 3) at the 95% level

The striking finding of our study is that nanoparticles exert their antiviral effects only when added after viral infection of the cells, which could be resulted in a significant decrease in viral titer. Post-exposure of H1N1 influenza virus with PEGylated ZnO-NPs at the concentrations of 75, 100, and 200 μg/mL could be led to 2.2, 2.4, and 2.8 log10 TCID50 reduction in virus titer when compared to the virus control, respectively (*P* < 0.0001), while the maximum concentration of ZnO-NPs (75 μg/mL) could resulted in 1.2 log10 TCID50 reduction (*P* < 0.0001). In our experiments, oseltamivir was used as a positive control for comparison of the anti-influenza activities of the test compounds. Moreover, the polyethylene glycol at its maximal non-cytotoxic concentration (200 μg/mL) could resulted in 0.7 log10 TCID50 reduction when compared to control (*P* < 0.0001) (Fig. 5).

**Fig. 6:**
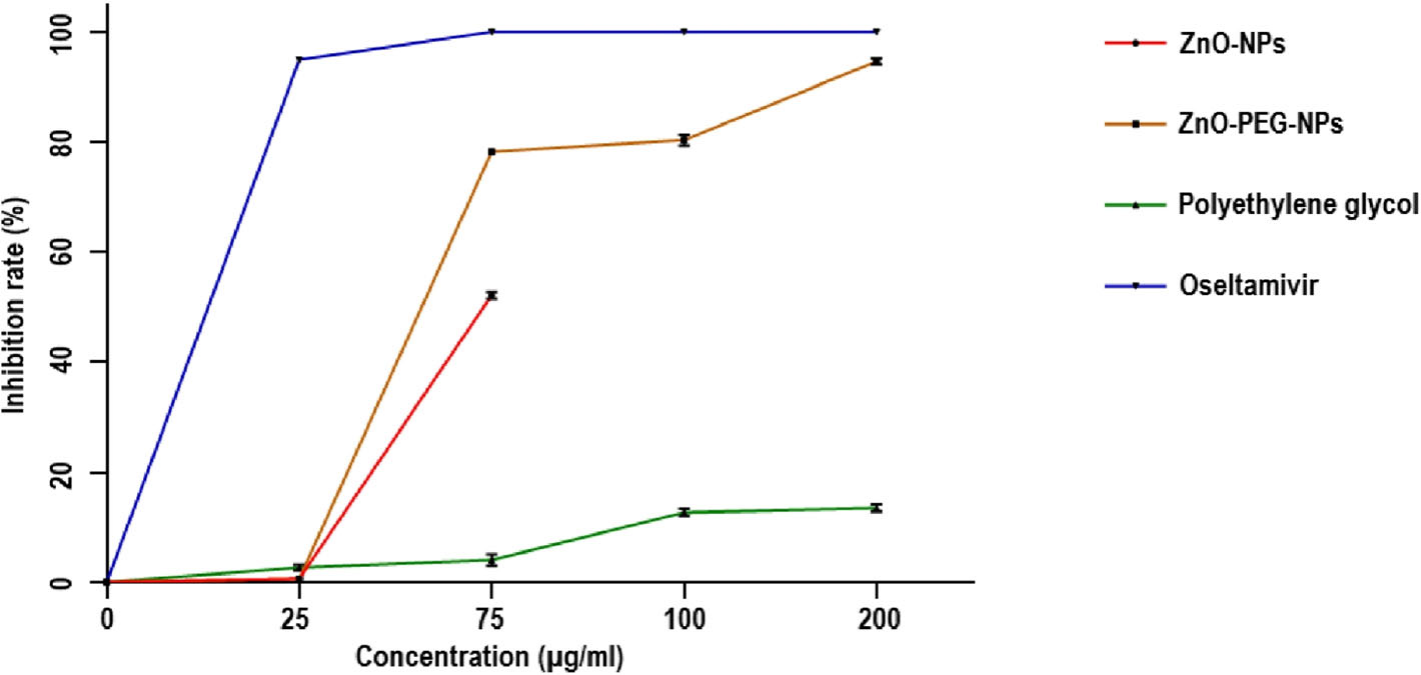
The inhibitory rates of the four compounds against H1N1 influenza virus determined by Real-Time PCR assay regarding to the post-exposure antiviral activity. According to the one-way ANOVA followed by Tukey’s multiple comparisons test, there were significant differences among the groups (*p* < 0.0001), except for ZnO-NPs vs. ZnO-PEG-NPs at the concentration of 25 μg/mL. Error bars represent the confidence interval for the mean (*n* = 3) at the 95% level

The antiviral activities of ZnO-NPs and ZnO-PEG-NPs against H1N1 influenza virus were further confirmed by quantitative Real-Time PCR. It was observed that the antiviral activity was in a dose-dependent manner, so that the ZnO-PEG-NPs at the concentration of 25, 75, 100, and 200 μg/mL led to inhibition rates of 0.6, 78.2, 80.3, and 94.6%, respectively. The inhibition rates were calculated based on the influenza viral loads. It is obvious that the anti-influenza activity of ZnO-PEG-NPs is greater than that of ZnO-NPs. The maximum antiviral effect of ZnO-NPs was obtained at the concentration of 75 μg/mL with the inhibition rate of 52.2% (Fig. 6). It is notable that the production of influenza virus was completely inhibited by oseltamivir at the concentration of 75 μg/mL. Furthermore, the inhibition rate of soluble polyethylene glycol was 13.5% at its maximal non-cytotoxic concentration (Fig. 6).

### Indirect immunofluorescence assay

**Fig. 7:**
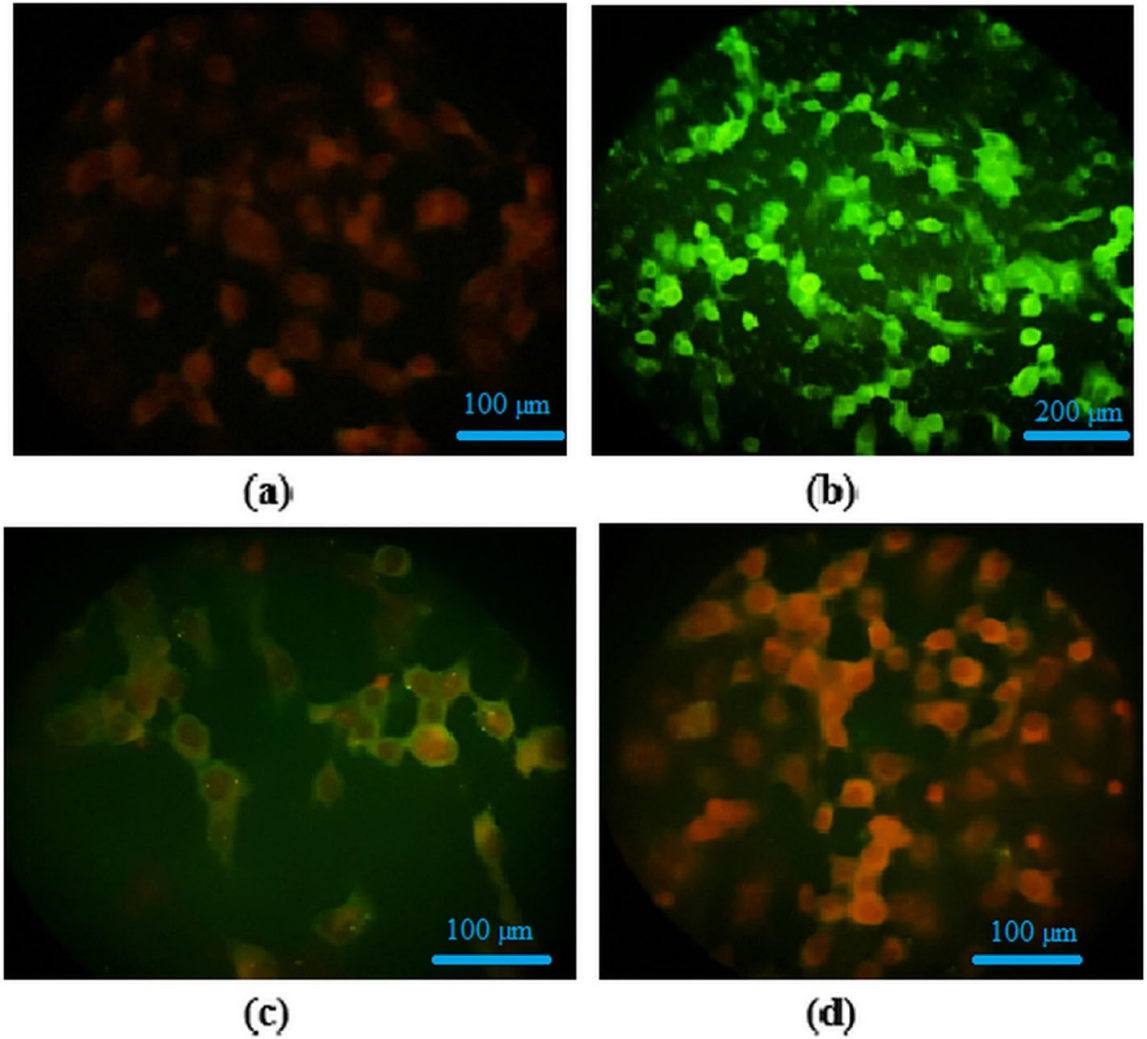
Immunofluorescence staining for detection of H1N1 influenza virus antigens in the MDCK-SIAT1 cells. (**a**) Cell control, (**b**) Virus control, (**c**) Infected cells treated with ZnO-NPs (75 μg/ml) and (**d**) 200 μg/ml ZnO-PEG-NPs at 24 h post infection

To study the inhibitory impact of nanoparticles on the expression of influenza virus antigens on the MDCK-SIAT1 cells surface, an indirect immunofluorescence assay (IFA) was performed. In this assay, we used the highest non-toxic concentration of ZnO-NPs and ZnO-PEG-NPs which showed the greatest antiviral effect in the previous experiments. In parallel, both negative and positive controls were also included. Figure 7 shows a substantial decrease in fluorescence emission intensity in influenza-infected cell treated with ZnO-PEG-NPs at the concentration of 200 μg/mL compared to the positive control, and with lower intensity in influenza-infected cell treated with ZnO-NPs at the concentration of 75 μg/mL.

## Discussion

The H1N1 influenza virus is a significant global public health threat with the potential to cause worldwide epidemics and pandemics. The current antiviral drugs against influenza are weakened due to their significant adverse effects and the increasing appearance of drug-resistant strains during the course of treatment [20] and so, development of new and effective anti-influenza agents is urgently required. Compared to the conventional treatments, there are several advantages to using nanoparticles for therapeutics such as effectiveness in lower concentrations, potent antiviral activity against drug-resistant viruses, cost-effective synthesis, and suitability for different coating types [16, 21]. In the current study, we used H1N1 strain, A/Puerto Rico/8/34 (H1N1) (PR8) as the virus model, and assessed the antiviral properties of ZnO-NPs and PEGylated ZnO-NPs on H1N1 virus. In order to achieve to this aim, we conducted a series of in vitro cell culture-based experiments.

Our results have shown that ZnO-PEG-NPs have a stronger antiviral effect along with lower cytotoxicity compared to ZnO-NPs, confirming that surface PEGylation of nanoparticles plays a key role in enhancement of antiviral activity against H1N1 influenza virus and reduction of cell cytotoxicity on MDCK-SIAT1 cells. Such results corroborate findings from our recent research in which we demonstrated that PEGylated ZnO-NP was associated with higher antiviral activity against herpes simplex virus type 1 (HSV-1) and lower cytotoxicity on Vero cell line [16]. In accordance with these findings, Martinez etal. Performed the MTT cell viability assay for assessment of cytotoxicity of ZnO-NPs and ZnO-PEG-NPs on MCF-7 breast cancer cells, and they demonstrated the higher cytotoxicity for bare ZnO-NPs compared to the PEGylated ZnO-NPs. In the previous studies, it has been revealed that ZnO-NPs produce Zn^2^+ ions and also, different types of reactive oxygen species (ROS) such as superoxides, hydroxyl radicals, and hydrogen peroxide which apparently damage lipids, proteins, carbohydrates and DNA, and finally lead to cell apoptosis [15]. As an explanation, it is proposed that the surface coating of ZnO-NPs with polymeric materials such as polyethylene glycol can lead to a significantly decrease in cytotoxicity through masking of nanoparticles and subsequently, preventing the release of Zn2+ ions and ROS.

The results have shown that the nanoparticles have inhibitory activities to suppress the proliferation of influenza virus when added 1 h after infection. As shown in the results, pre- and co-exposure of cells to nanoparticles did not result in any decrease in the titer of H1N1 influenza virus. These findings indicate that the nanoparticles do not induce antiviral state on MDCK-SIAT1 cells, and also, do not occupied specific receptors involved in attachment and entry of influenza particles into the host cells. These experimental results show that the nanoparticles target and interfere with the some stages in the life cycle of the influenza virus which occur after viral adsorption and internalization by the cells.

Although, the results of antiviral assay revealed that inhibitory potential of ZnO-NPs and ZnO-PEG-NPs against H1N1 influenza virus was only documented in post-exposure antiviral assay, antiviral activity during the pre- and co-exposure of cells to the nanoparticles cannot be completely ruled out. This possibility arises from different incubation times of nanoparticles and virus. In the post-infection setup, the nanoparticles were incubated with infected cells and viruses for 48 h, whereas it was shorter (≤1 h) for pre- and co-treatment assays. Here, incubation time is a key factor and can dramatically influence the cellular uptake of nanoparticles.

It has been reported that due to increase in surface hydrophilicity, PEGylation leads to reduced cellular uptake of nanoparticles [22, 23]. On the other hand, influenza viruses carry out their transcription and replication entirely inside the cell [24]. Here, the question is that how the PEGylated ZnO nanoparticles can exert the higher antiviral activity than the bare ZnO nanoparticles? This obvious discrepancy can be rationalized by the explanation that PEGylation of nanoparticles using the severe mechanical ball milling in our study led to production of smaller particle size. It should be noted that smaller nanoparticles are more likely to be passively internalized by cells [25].

ZnO-PEG-NPs have been shown to have higher antiviral potency than the bare ZnO-NPs at the same concentrations. The difference can be justified by several explanations. Our findings demonstrated that soluble polyethylene glycol at its maximal non-cytotoxic concentration (200 μg/ml) had slightly antiviral activity on influenza virus (with inhibition rate of 13.5%). As an explanation, the higher antiviral activity of ZnO-PEG-NPs than the bare ZnO-NPs can be attributed to the surface PEGylation of ZnO nanoparticles. On the other hand, PEGylation of nanoparticles using the severe mechanical ball milling in our study resulted in production of nanostructures with smaller particle size. It should be noted that decreasing in particle size of nanoparticles leads to: (1) increased surface area to volume ratio; (2) facilitated diffusion of particles into cells; and (3) decreased agglomeration and increased rate of dissolution properties.

Over recent years, few studies have been conducted to investigate the inhibitory effects of various nanostructures on influenza virus infection. In the most recent work, Kumar et al. assessed antiviral activity of Fe3O4 nanoparticles (IO-NPs) against PR8-H1N1strain, and they proposed the IO-NPs as the potent influenza virus inhibitor with 8 fold decrease in viral RNA [26]. In another study by Lin et al., antiviral properties of selenium nanoparticles (SeNPs) and zanamivir modified selenium nanoparticles (Se@ZNV) against H1N1 influenza virus investigated, and their results indicated that Se@ZNV has a higher antiviral activity compared to the SeNPs and zanamivir alone [27]. Similarly, Li et al. conducted a study to investigate antiviral capabilities of SeNPs and oseltamivir surface-modified SeNPs (Se@OTV) against H1N1 influenza virus [28]. Their findings showed that Se@OTV is associated with higher antiviral activity and has less toxicity. Interference with influenza virus life cycle by inhibition of hemagglutinin and neuraminidase activities was suggested as a possible mechanism.

During the last stage of the influenza virus replication cycle, newly assembled viral particles should be released from the cell surface. In this step, influenza’s neuraminidase enzyme cleaves the attachment between hemagglutinin on the progeny virus and sialic acid receptor on the host cell. Oseltamivir and zanamivir are sialic acid analogues and neuraminidase inhibitors which prevents this cleavage step, and interfere with the release of progeny influenza virus from infected host cells and subsequently, prevent the progression of infection [29, 30]. Since that in the studies conducted by Lin et al. and Li et al., oseltamivir and zanamivir modified selenium nanoparticles were used for evaluation of anti-influenza activity, the possible antiviral mechanism could be inhibition of hemagglutinin and neuraminidase activities.

## Conclusions

Our study was the first research which examined the inhibitory effects of ZnO-NPs on H1N1 influenza virus. The results showed that PEGylated ZnO-NPs have a higher anti-influenza activity along with lower cytotoxicity compared to bare ZnO-NPs, suggesting that surface PEGylation of nanoparticles can be effective in enhancement of antiviral activity against H1N1 influenza virus and reduction of cell cytotoxicity on MDCK-SIAT1 cells. Our in vitro experiments also demonstrated that the nanoparticles have inhibitory properties against influenza virus only after viral entry into the host cells. The future studies can be designed to explore the exact antiviral mechanism of the nanoparticles using more developed techniques such as transmission electron microscope (TEM), as well as to examine the pattern of influenza virus gene expression in the presence of the nanoparticles.

## Data Availability

Not applicable.
